# Adapting a perinatal empathic training method from South Africa to Germany

**DOI:** 10.1186/s40814-018-0292-6

**Published:** 2018-06-19

**Authors:** Caprice Knapp, Simone Honikman, Michael Wirsching, Gidah Husni-Pascha, Eva Hänselmann

**Affiliations:** 1 Department of Health Policy and Administration, Pennsylvania State University, State College, USA; 2 Department of Psychotherapy and Psychosomatic Medicine, Faculty of Medicine, University of Freiburg, Freiburg im Breisgau, Germany; 3 Perinatal Mental Health Project, Alan J Flisher Centre for Public Mental Health, Department of Psychiatry and Mental Health, University of Cape Town, Cape Town, South Africa

**Keywords:** Maternal mental health, Experiential learning, Work-based learning, Empathy in maternal care staff, Self-care in maternal care staff, Intercultural adaptation, Survey development, South to north transfer

## Abstract

**Background:**

Maternal mental health conditions are prevalent across the world. For women, the perinatal period is associated with increased rates of depression and anxiety. At the same time, there is widespread documentation of disrespectful care for women by maternity health staff. Improving the empathic engagement skills of maternity healthcare workers may enable them to respond to the mental health needs of their clients more effectively. In South Africa, a participatory empathic training method, the “Secret History” has been used as part of a national Department of Health training program with maternity staff and has showed promising results. For this paper, we aimed to describe an adaptation of the Secret History empathic training method from the South African to the German setting and to evaluate the adapted training.

**Methods:**

The pilot study occurred in an academic medical center in Germany. A focus group (*n* = 8) was used to adapt the training by describing the local context and changing the materials to be relevant to Germany. After adapting the materials, the pilot training was conducted with a mixed group of professionals (*n* = 15), many of whom were trainers themselves. A pre-post survey assessed the participants’ empathy levels and attitudes towards the training method.

**Results:**

In adapting the materials, the focus group discussion generated several experiences that were considered to be typical interpersonal and structural challenges facing healthcare workers in maternal care in Germany. These experiences were crafted into case scenarios that then formed the basis of the activities used in the Secret History empathic training pilot. Evaluation of the pilot training showed that although the participants had high levels of empathy in the pre-phase (100% estimated their empathic ability as high or very high), 69% became more aware of their own emotional experiences with patients and the need for self-care after the training. A majority, or 85%, indicated that the training was relevant to their work as clinicians and trainers, that it reflected the German situation, and that it may be useful ultimately to address emotional distress in mothers in the perinatal phase.

**Conclusions:**

Our study suggests that it is possible to adapt an empathic training method developed in a South African setting and apply it to a German setting, and that it is well received by participants who may be involved in healthcare worker training. More research is needed to assess adaptations with other groups of healthcare workers in different settings and to assess empathic skill outcomes for participants and women in the perinatal period.

## Background

Perinatal mental health disorders such as depression and anxiety are prevalent in 10–13% of women from high-income countries (HIC) and 15–19% from low- and middle-income countries (LMIC). Factors associated with perinatal depression include younger maternal age, lower educational status, lack of social support, unintended pregnancy, previous mental illness, intimate partner violence and socioeconomic disadvantage [1, 2]. These disorders are associated with substantial morbidity for mothers and their offspring, exerting impacts at neuroendocrinological, epigenetic, behavioral, and social levels [3, 4]. In extreme cases, these disorders can lead to suicide. In HIC, suicide is rated among the leading causes of maternal mortality [5] while contributing smaller, but notable proportions of maternal deaths in LMIC [6].

Efforts to improve perinatal mental health care have focused on policy, delivery, and capacity building. A key recommendation for addressing the challenges of perinatal mental health is to integrate mental and maternal health care [7, 8]. Successful integration of mental health and maternal health care has been demonstrated in both LMIC [9] and HMIC [7, 10–13]. Rahman et al. note that challenges for successful integration include, among others, to “1) enhance collaboration between maternal and child health and mental health programs, researchers, and practitioners […]; 2) address stigma related to mental illness that could impede integration […]; 3) increase capacity in LMIC by creating regional centers for mental health research, education, training, and practice that incorporate the views and needs of local people […], 4) strengthen the mental health component in the training of all health care personnel […]” [8].

The Perinatal Mental Health Project (PMHP) of the University of Cape Town has been operating a model of integrated mental health care within primary level maternity facilities at three units in Cape Town, South Africa. Non-specialist counselors provide psychological treatments that include elements of behavioral activation, problem solving, and interpersonal therapy as well as nonspecific supportive counseling elements. The service has demonstrated promising results in terms of service utilization and symptom reduction [10]. To support this model and to address several of Rahman’s challenges, the PMHP developed and refined over the course of 12 years, a participatory empathy training method for all types of healthcare workers called *Secret History* [14]. The aims of the training are to reduce high levels of disrespectful care among maternity healthcare workers [15, 16], increase their knowledge of maternal mental illness, and improve their empathic engagement skills so that they may be more responsive to the emotional needs of vulnerable mothers. It is hypothesized that the training will support the success of integration of mental and maternal health care. The method has been adapted and used nationally with healthcare workers in training, practice, and postgraduate instruction.

The value of empathic engagement elements in the mental health setting was well described in a recent systematic review and meta-analysis. It was reported that psychological treatments for common mental disorders in a range of primary care settings in LMIC, and delivered by non-specialist providers, had moderate to strong effects in reducing the burden of these disorders. Notably, 12 out of 27 eligible studies took place in maternal or reproductive service settings. A key finding pertains to the analysis of nonspecific therapeutic elements, called *engagement elements*, which includes collaboration, empathy, active listening, normalization, and involving partners or family. These elements predicted trial effectiveness independently of and comparably to specific elements, e.g., interpersonal, cognitive, and behavioral [17]. Although lessons from LMIC settings may be beneficial for the delivery of more efficient integrated perinatal mental healthcare, it is not clear how these interventions may be adapted for HIC. To our knowledge, there is a lack of evidence on how interventions may be adapted from LMIC to HIC.

The aims of this paper is (1) to describe an adaptation of the Secret History empathic training method from the South African to the German setting and (2) to assess the acceptability of the adapted training among trainers.

### Setting

Germany was chosen as the setting for several reasons. First, Germany has consistently had the highest success in treatment of psychosocial and psychosomatic issues of 30 European countries assessed in a comparison conducted by the Economist Intelligence Unit since 2014 [18]. However, there remains a need for improvement of maternal mental health care in Germany. Here, maternal mental health care is still in the early stages of development and focuses particularly on the mother-infant relationship [19, 20]. Second, the project was facilitated by a prior working relationship between the University of Cape Town and Freiburg University as part of the Pan Institute Network for Global Health. Adaptation, delivery, and evaluation of the Secret History empathic training method took place at the Medical Center at the University of Freiburg in Germany.

### Intervention

The Secret History draws from the critical education principles of Paulo Freire [21] and the associated techniques used in Theater of the Oppressed developed by Augusto Boal [22]. The Secret History method includes two group role play exercises, didactic learning, and debriefing. Each is described below. More information about the process of adaptation and training details is provided elsewhere [23].

After introductions and an “ice-breaker” involving physical activity, the training begins with a group role play exercise. In the first role play, the participants are divided in two halves, seated and facing each other across the room. Two facilitators invite the participants to discover the secrets of two people: a patient and a healthcare worker who each experiences typical scenarios of adversity and emotional distress in their work or personal lives. Each group is encouraged to “become” the patient or healthcare worker. The facilitator each align themselves with a group and reveals to all participants, in stages, the background narrative of each role using the first person plural to support each group’s identification with their role.

At each stage, the actors are encouraged to communicate with each other in a naturalistic, but disrespectful way as if they were engaging in a real-world setting. Before each new stage of the narrative, each group is asked to suspend their interactions and identify their feelings and needs, which are recorded on a white board by their facilitator. After several iterations, participants physically switch sides and "become" the other role. The narrative and disrespectful engagement continues until the groups are asked to return to their original seating and return to their own selves. Debriefing follows where the experience is discussed and analyzed. Participants are encouraged to reflect on the similarities between the patient’s and healthcare worker’s feelings and needs, yet how their disconnection may fuel a vicious cycle of disrespect between the pregnant woman and the healthcare worker. Facilitators evoke the analogy of each character’s secret history being revealed like baggage or mental health challenges brought into the relationship. Through the frameworks of professionalism and self-care, discussion unfolds about the care givers’ roles and responsibilities with respect to their own and their patients’ mental health challenges.

Next, a didactic learning component includes an introduction to six empathic skills including: creating a safe space, verbal and non-verbal communication, reflecting feelings, affirming, getting feedback, and knowledge sharing.

Another group role play exercise is then conducted, again using typical scenarios of life challenges for each character. The participants are configured in a fishbowl shape with two participants in the middle. These two take on the patient and care provider roles, as individuals, and demonstrate the six skills. The rest of the group observe their interactions as they continue through a new case narrative that includes two characters. As the narrative continues, the two actors are interchanged with different participants giving as many in the group as possible a chance to demonstrate the learned skills. A facilitator interrupts the role play to highlight and affirm where the actors are demonstrating the skills well, noting different, but equally valid, styles of empathy. Guidance is provided to participants who may struggle with the role play. The training ends with a final debriefing session where participants are encouraged to reflect on the differences in their experiences between the two role play exercises and what they learnt from the training as a whole.

The facilitators end the training with an exploration of matters pertaining to self-care. It is noted that disrespectful care may result from, but also contribute to emotional fatigue or a lack of feeling cared for. While providing empathic care may also have attendant consequences of fatigue or burnout. Thus, self-care is highlighted as a preventive and self-maintenance process that is critical to effective care provision, especially when patients are experiencing emotional distress.

## Methods

The Secret History method was adapted, a pilot training was conducted, and this training was evaluated. Each of these components are described below.

### Adaptation

A focus group, conducted by a trained psychologist together with author EH in German, was used to inform the adaptation process. Participants (*n* = 8) from relevant clinical departments, such as obstetrics, gynecology, and midwifery, and students of these subjects, were invited by email to participate. Staff supervisors helped in recruiting participants. The eight participants were four staff from the obstetric ward, the head of the local midwifery school, two midwifery students, and a local psychosomatic care trainer in gynecology. A discussion guide was used and structured notes were taken. The discussion was audio taped and the results were analyzed.

Given that the purpose of the focus group was to use the information to adapt the training materials, themes and descriptions of challenging cases were noted by three investigators independently. A thematic analysis based on a social constructionist epistemological framework was conducted on the written material. New German case scenarios were then created for Secret History training, replacing the South African cases. Adapting training materials across cultures is critical to improving local effectiveness [24, 25]. Existing literature also indicates that omitting adaptation “would threaten program efficacy, despite high fidelity in program implementation” [26]. Results were validated by member checking giving the participants the opportunity to comment on the new case scenarios.

### Pilot training

Invitations were sent to the officer for Innovation and Quality of Care and the obstetrics ward at Freiburg University Hospital, the midwifery school, and the nursing school via email or made in-person for a pilot training, which was held in July 2016. All participants were chosen from partner institutions and therefore represent a non-random purposeful sample.

The pilot training sought to (1) deliver the training, (2) determine the potential for the participants to become trainers in the future, and (3) evaluate the training and its adaptation. The diverse group (*n* = 15) included professional trainers from perinatal health and mental health units, curriculum developers in nursing and midwifery, psychologists with experience in psychosomatics, and obstetrics staff.

The two trainers conducted most of the session in English as SH was unable to speak German and the majority of the participants were able to converse comfortably in English. MW and GHP were available for participants to use as interpreters where this was required. The final design of the pilot followed the original Secret History’s structure.

### Evaluation

A survey was developed to evaluate the training. This was critical not only to determine the results of the pilot training, but so that future training sessions can be evaluated consistently. Pre- and post surveys were developed based on a review of the literature on perinatal mental health and empathy. Although there were existing survey tools on empathy, most were patient-reported or clinician-reported and used to evaluate provider-patient interactions [27]. Although these were not applicable in our training of health care workers, the surveys informed what domains and constructs should be included. Because the aim of this study was to evaluate empathy training with health care workers, we also reviewed literature on health care worker training. Our survey design was also informed by experiences of the Secret History developer (SH) and expertise in survey design and analysis (CK).

A pre-post survey was created, which included questions on knowledge on empathy, self-reported empathy, and attitudes towards patients. Items used a 5-point Likert scale, seven scaled response categories indicating frequency or true or false options, e.g., “Ich finde es manchmal schwierig, die Dinge aus der Perspektive der Mutter zu sehen” (I sometimes find it difficult to see things from the mother’s point of view) with a 5-point Likert scale; “Ich versetze mich in die Lage meiner Patientinnen” (I put myself in my patient’s shoes) with seven options indicating frequency from 1 (never) to 7 (daily); and “Unter Mitgefühl und Empathie versteht man zwei unterschiedliche Konzepte” (compassion and empathy are not the same concepts) with true or false options. The post survey also included a section specific to the training, e.g., “Der Kurs verfügte über eine gute Mischung von theoretischen Einheiten und praktischen Übungen” (the course had a good mix of didactic learning and exercises) with a five-point Likert scale and “Was ist das Wichtigste, das Sie in diesem Kurs gelernt haben” (what is the most important thing you learned in this training) or “Welche Verbesserungsvorschläge haben Sie für diesen Kurs” (how would you suggest the training be improved) as an open-ended question. Prior to the actual pilot training, the pre-post survey included 73 closed and 5 open-ended items. Subsequent item analysis was conducted to reduce the number of items. The final survey includes 19 closed 4 open-ended items. The survey is available upon request from the first author.

IBM SPSS Statistics Version 24 was used in the analysis. Self-estimation items with regard to their empathic ability were averaged to yield an output parameter with seven levels, where a seven indicates high empathic abilities. Item reliability was tested using Cronbach’s alpha. Pre-post changes in items values were tested using a two-sided paired *t* test.

## Results

### Adaptation

The participants were predominantly women, experienced practitioners (average 32 years of experience), and trainers of health professionals. Several themes and typical challenging situations emerged that were used to adapt the training materials.

Care of migrant women emerged as a major theme. Issues that are challenging for maternal staff trying to deliver empathic care include language and cultural misunderstandings, specific clinical issues such as female circumcision, and psychosocial strain on migrant women. Participants noted that, “it is refugee women who bear the largest burden.”^1^ One participant said, “Many women – migrant women, but also in the German population; we are talking about one in five women – are sexually traumatized and naturally don’t tell you before, but it comes out during birth.”

Another theme that emerged was communication and cultural issues pertaining to expectant fathers. Participants referred to their experience that, “husbands sometimes turn aggressive [towards nurses or midwifes], because they are frightened and overwhelmed” or in the case of migrant husbands “they are not used to seeing women work so independently.” A typical situation was described where a migrant husband became aggressive and yelled at a nurse who was consulting the mother about breastfeeding. Participants noted that, in these situations, they could be “truly frightened.” With regard to empathic communication with the mother, participants stressed that using non-technical language was critical. One participant said, “When I talk to the doctor in the hallway, I use medical terminology, but when I enter the mother’s room, I have to talk in a different way.”

A final theme emerged around self-care. Job-related stress contributes to participants’ difficulty delivering the empathic care they would like to provide. Nurses and midwives articulated, “[With view to challenges in daily work], the interdisciplinary conflicts with the medical doctors are in the foreground for me.” Also, “the expertise one has is ignored and they [the doctors] don’t listen to us.” Participants agreed that improvement of obstetric care should draw on a multidisciplinary approach “since so many different professions are involved, especially in a university medical clinic.” More detailed results of the focus group discussion are reported elsewhere [23].

The South African version of the Secret History includes cases pertaining to mothers who were HIV positive, adolescent, or had experienced rape and domestic violence. Drawing on the focus group discussion, the German version of the method included for the first case, a scenario of an isolated, pregnant adolescent woman. For the second case, the scenario was of an immigrant woman in labor, accompanied by a frightened and aggressive husband. The adapted scenarios also included the theme of dysfunctional power dynamics between different cadres of healthcare staff.

### Pilot training

A 6-h pilot training was held in July 2016. Among the final 15 participants were three midwives, one nursing instructor, two physicians, four psychologists, two administrators, a nursing specialist for obstetric care, and two other local stakeholders.

After an ice-breaker, the group role play embodying disrespectful care used the case of an adolescent woman in labor. The debriefing elements in the South African version focused predominantly on burnout issues and healthcare worker personal experiences of disrespect and emotional distress. For the German adaptation, the debriefing component, group members expressed that they “felt the other side did not understand their needs and feelings” that the “other side frustrated me and ignored me.” Participants debated whether the case scenario was reflective of real clinical situations in Germany. The physicians were not aware of similar real scenarios while the remaining participants indicated that the quality of the narrative was in fact realistic for the German setting.

Thereafter, a briefer version of the didactic session took place describing the empathic skills. This element was shortened because the group indicated familiarity with the material as many had experience training others in basic counseling or empathic methods.

The second role play exercise of a pregnant immigrant woman and her argumentative husband (neither of whom spoke much German) was conducted using the original fishbowl method with most participants having turns to play the healthcare worker. The debriefing focused on issues of cultural barriers and xenophobia and participants noted that the scenario was reflective of reality in the German obstetric setting. The group also discussed the often-undermining and hierarchical relationships between nurses, midwives, and doctors in Germany. The group considered that the most effective empathic activities were speaking in a low voice, providing a non-intrusive touch such as rubbing the back, addressing the husband’s concerns in a location away from the laboring mother, and acknowledging the husband’s needs and concerns in a respectful manner. Like the South African version, there was discussion about the barriers to providing empathic care, the benefits of providing this care and on self-care for the healthcare worker.

### Evaluation

The pre-survey results indicated that pilot participants had high levels of empathy prior to the training. Reliability testing of the empathy-related items yielded a Cronbach’s alpha of 0.8 indicating inter-item correlation and allowing for item averaging. All participants estimated their empathic abilities high or very high, (6 or 7, on a 7-point scale). As noted earlier, the participants were mid- to senior level staff so this was somewhat expected. For example, the majority indicated that they “put themselves in the patient’s shoes” or “I give my patients enough time as they need to speak”(see Table 1).

**Table 1:** Frequency analysis of empathy-related items in the pre-survey, options from 1 = never to 7 = daily

	Valid					Missing	Total
	Regular (4)	Often (5)	Very often (6)	Daily (7)	Total		
I am friendly and caring with patients			3	8	11	2	13
I give patients as much time as they need to speak			6	5	11	2	13
I pay attention to what the patient finds difficult			3	8	11	2	13
I make sure I give answers to my patient’s questions and provide information that is clear			3	8	11	2	13
I fully understand my patient’s concerns	1	2	7	1	11	2	13
I put myself in my patient’s shoes	1	1	3	6	11	2	13

Likewise, items relevant to the participants’ understanding of their own role in care and the perceived relevance of empathic care had a strong ceiling effect. When comparing these items pre-post in a paired *t* test, no further change towards more empathic care were detected (*p* values of pre-post tested items ranged from 1 to 0.167).

When asked what patient behaviors made it challenging to be empathic, there was little agreement (4 out of 13 valid responses) among the participants about “not showing up for appointments” and “not taking personal responsibility for their health.” Most participants (7 out of 11 valid responses) agreed that challenges to empathic care included clients’ “having personal problems” whereas only one participant found it especially difficult to care for patients “not speaking the native language” (see Table 2).

**Table 2:** What characteristics do you think make it hard to be empathetic towards patients?

	Valid					Missing	Total
	Strongly agree (1)	Agree (2)	Neutral (3)	Disagree (4)	Strongly disagree (5)		
Patients who do not show up for appointments		4	3	1	2	3	13
Patients who do not take personal responsibility for their health	1	3	2	3	2	2	13
Patients who have many personal problems	3	4	3		1	2	13
Patients who cannot speak the native language		1	1	5	4	2	13

The post survey asked specifically about the training itself. Responses included that the method was “relevant to my job”, “had a good mix of didactic training and exercises”, “the training uses a novel approach” and “it seemed like it represented typical German patients/encounters”. Some areas where there was less agreement included concern about being able to teach the method to others (six participants agreed, six were indifferent, one disagreed) and the short duration of the training (four found it too short, four were indifferent, five did not think it was too short) (see Table 3).

**Table 3:** Training course feedback

	Valid					Missing	Total
	Strongly agree (1)	Agree (2)	Neutral (3)	Disagree (4)	Strongly disagree (5)		
The course had a good mix of didactic learning and exercises.	11	2					13
The training did not seem relevant for my job.				2	11		13
The scenarios seemed like they represented typical patients/encounters.	3	3	5	1		1	13
The training was too short.	1	3	4	5			13
I feel confident that I could train others in the Secret History method.		6	6	1			13

An open-ended survey question asked for the most important thing the participants learned from the training. The most common answer was the importance of the awareness of one’s own emotional experience in empathic engagements. Many participants also expressed surprise in the debriefing session at the corresponding feelings of characters in both roles, e.g., calmness in the health worker bringing about calm in the patient. The most frequent suggestions for how to improve the training was to have more time for role playing and to give more guidance to participants regarding how they may facilitate such a training themselves. Eleven out of 13 participants thought that the training will impact them personally as well as professionally. One participant wrote, “Empathy and the ability to enter into relationships have to be maintained like precious tools = self-care!!”.

## Discussion

There is an established body of evidence for the positive impact of psychological interventions on perinatal mental health in HIC [28, 29] and a growing body of evidence for this in LMIC [30]. For the latter, the use of lay workers to deliver simple psychological interventions and supportive counseling is emerging [30–32]. However, there is a paucity of evidence regarding utilization of maternity staff to employ nonspecific engagement approaches that may be used to positively impact patients’ emotional distress [17].

Since most existing studies on empathy education in nursing are set in North America or the UK [33], our study broadens the geographic and cultural scope of the empathy training literature.

There is lack of literature on work-based interventions to strengthen self-care in nurses and midwives [34]. Of 17 studies included in a review on empathy education in nursing training, only one included a self-care component [33]. At the same time, it is well documented that empathic engagement bears the risk for providers of over-involvement, increased depression [35], and secondary traumatic stress [36]. The Secret History training method, in both the original and adapted versions, addresses this need, and follows Brunero’s suggestion that empathy education should highlight the role of self-awareness when being empathic [33].

Although there is increasing evidence in the literature for successful interventions developed in LMIC, there is little information on how those interventions might be utilized in HIC. Part of this could be due to the challenges in funding research and publishing results. Collyer discusses the inequities of publishing in the Global South [37]. Even when interventions are known to those in HIC, as in our study, the process of adaptation and utilization is uncommon. Literature about translation and cultural competency exist but we found none about adaptation methods from LMIC to HIC. The methods we used may be replicated in the future and increase the contributions of LMIC.

The generalizability of this work is limited by the single context chosen for the adaptation. Certainly, there may have been different outcomes should this work have been conducted in other HIC countries, or other settings within Germany. However, Germany has been at the forefront of mental health in Europe [18]. While it may be argued that this makes our results less generalizable, emerging migrant issues, with their attendant social challenges, are widely prevalent and are certainly applicable to many other HIC. Increased migration will necessitate adaptations of healthcare delivery, particularly in mental health and maternal health, which are particular areas of need for immigrants, refugees, and internally displaced persons [38, 39]. Our results may have been biased by the participants who took part in the pilot testing, most of whom were experienced clinicians or healthcare trainers with high levels of empathy in the pre-survey. It is uncertain whether or how less experienced healthcare workers or students, outside of a major academic medical center would have benefitted more from the training.

## Conclusion

Despite these limitations, our study has demonstrated that a contextually adapted version of the Secret History empathic training method for perinatal mental health is acceptable and useful in a German setting. As empathic engagement has demonstrated positive impact on mental health outcomes for common mental disorders in perinatal populations in LMICs, future research should explore whether in HIC, empathic training for maternity care staff, will lead to a reduction in the effects of common perinatal mental disorders in their clients.

## Abbreviations

HIC: High-income countries
LMIC: Low- and middle-income countries
PINGH: Pan Institution Network for Global Health
WHO: World Health Organization

## Footnotes

1 Quotes from the discussion were translated from German to English by the last author EH.
